# The Relationship Between and Correlates of Problematic Sexual Behavior and Major Mental Illness

**DOI:** 10.3389/fpsyg.2021.719082

**Published:** 2022-01-04

**Authors:** Heather M. Moulden, Casey Myers, Anastasia Lori, Gary Chaimowitz

**Affiliations:** ^1^Forensic Psychiatry Program, St. Joseph’s Healthcare Hamilton, Hamilton, ON, Canada; ^2^Department of Psychiatry and Behavioral Neurosciences, McMaster University, Hamilton, ON, Canada; ^3^Department of Psychology, Neuroscience and Behavior, McMaster University, Hamilton, ON, Canada

**Keywords:** sexual abuse, mental illness, psychosis, sexual offending, forensic

## Abstract

While research has consistently found that general distress and psychopathology are not predictive of sexual recidivism, examination of specific syndromes and their relationship to offending has revealed a potentially more complicated relationship. One proposed mechanism for the mixed findings with respect to major mental illness and sexual offending may be the confound of neurological injury. As identified in Mann et al. (2010) work on psychologically meaningful risk factors, mental illness represents an area in need of more study given the indirect influence it may exert on risk. To this end, the current paper summarizes the study of the relationship between neurological injury, psychosis and problematic sexual behavior among two Canadian samples of forensic and civil psychiatric patients. In the first study we observed higher than expected rates of sexually-themed psychotic symptoms (45%) and problematic sexual behavior (PSB; 40%) among a combined group of forensic and civil psychiatric patients (*n* = 109). Indeed 70 percent of those individuals who engaged in PSB endorsed sexually-themed psychotic symptoms. While comorbidity is common amongst this group, brain injury appeared to represent a specific liability. Compared to those who did not engage in PSB, those who did were almost 4x (OR = 3.83) more likely to have a documented history of brain injury (e.g., traumatic and acquired brain injury, including fetal alcohol syndrome). In the second study we sought to replicate this finding in a larger forensic sample of 1,240. However, the recorded rates of brain injury were significantly less, such that no relationship to PSB was observed. Based on the mixed findings to date, including our own data, questions remain about the nature of a potential shared vulnerability for psychosis and PSB previously postulated. Among psychiatrically complex individuals who engage in PSB, understanding etiology and links to risk are helpful, but perhaps more importantly is attention to the mechanisms through which symptoms confer risk (e.g., problem solving, sexual disinhibition, social/intimacy deficits) and how best to treat and manage them.

## Introduction

Deinstitutionalization and an overburdened health care system have contributed to the steadily increasing rates of mental illness in prisons and community corrections, Abracen et al. (2014, 2016). Previously those individuals diagnosed with major mental illness (MMI) were thought to belong strictly to the forensic psychiatric realm, but research shows that in fact, many individuals who are found not criminally responsible or unfit to stand trial have previous criminal charges and convictions, with a significant proportion having served custodial sentences (Moulden et al., 2014; Skeem et al., 2014; Moulden and Myers, 2017; Chaimowitz et al., 2021). The fluidity between systems suggests that some knowledge can be shared about individuals with mental illness, regardless of how their behavior is adjudicated. However, the specific contribution of mental illness to questions of etiology and risk has been hotly debated. In the realm of sexual violence, broad definitions of mental illness, or even “personal distress” have been considered in meta-analyses, but have failed to emerge as a relevant risk factor for sexual recidivism (Bonta et al., 2014). However, not surprisingly, more nuanced studies with specific attention to more serious or profound psychiatric disorder, have revealed evidence of a relationship between MMI and sexual offending (for a comprehensive review see Moulden et al., 2020).

Major mental illness describes severe and chronic psychiatric disorders, characterized by perceptual disturbance, cognitive and behavioral instability, social dysfunction, and potentially significant functional disability (Moulden and Marshall, 2017). The term typically refers to psychotic disorders or those disorders with psychotic features, which include symptoms that not only interfere with perceptions of reality, such as hallucinations and delusions, but also impact on social, motivational, and cognitive processes according to the *Diagnostic and Statistical Manual of Mental Disorders*, 5th edition [American Psychiatric Association (APA), 2013]. Psychotic illnesses are also characterized by impairment in the experience and expression of emotion, cognitive deficits, poor insight, neurological deficits, aggression, and problems inferring the emotions and intentions of others [e.g., theory of mind—American Psychiatric Association (APA), 2013]. It is easy to see various ways in which such mental illness could contribute to problematic sexual behavior (PSB) and undermine treatment efforts designed to address it and thus prevent reoffending. It is precisely these aspects, causation and treatment amenability that Mann and her colleagues referred to when contemplating what constitutes a psychologically meaningful risk factor for sexual reoffending (Mann et al., 2010).

In their seminal paper, Mann et al. (2010) proposed a new way to understand risk factors, referring to the concept instead as *psychologically meaningful risk factors*. In doing so they attempted to focus on individual propensities as “enduring characteristics that leads to predictable expressions of thoughts, feelings, or behaviors. Although propensities are characteristics of individuals, these propensities can also be recognized by individuals’ transactions with others and the environments in which they live” (Mann et al., 2010, p. 194). In their paper the authors sought to identify and synthesize the evidence for candidate psychologically meaningful risk factors. In addition to theoretical and clinical support, these factors also needed to demonstrate evidence that they predict sexual recidivism. MMI falls within the third of five tiers, described as “unsupported, but with interesting exceptions.” In their analysis, the overall effect of MMI is determined to be small (*d* = 0.24) based on the available meta-analytical data at the time. However, the interesting exception was noted in the large effect (*d* = 0.90) reported in the Swedish record study, which provided support and a rationale for ongoing study (Långström et al., 2004).

One of the issues that has contributed to the discrepant findings regarding MMI and sexual offending, has been methodological (see Moulden and Marshall, 2017). Definitions of mental illness, and reliance on incarcerated samples, excluding forensic patients and those with non-adjudicated PSB (Guidry et al., 2008), has potentially diluted the effect for individuals with a primary psychotic illness. This is where the present study picks up. In an attempt to address the theoretical questions, about how and why psychosis and PSB may be linked (Drake and Pathé, 2004; see Moulden et al., 2020), it seemed to make sense to include those who possess the very phenomenon of interest, but have heretofore been unevenly represented in the research. In the course of this research we had three main questions: (1) what is the rate of PSB among those with psychosis, (2) to what extent is psychosis sexually-themed, and (3) is there a relationship with brain injury. This third question emerged from research showing that traumatic/acquired brain injury and other related congenital neurological deficits are overrepresented in both psychiatric and justice-involved groups (Shiroma et al., 2012), and particularly so, among individuals who engage in PSB. Furthermore, based on research suggesting shared vulnerability between psychosis and paraphilias, as well as clinical observation and our own clinic data, this was an opportunity to explore if brain injury has a role to play in the ongoing study of MMI and PSB.

In order to provide some context, neurological explanations are an important aspect of etiological theories of both sexual interest and socially deviant behavior. For example, Ward and Beech’s (Ward and Beech, 2006) Integrated Theory of Sexual Offending (ITSO) attempted to explain the onset, development and maintenance of sexual offending as arising from the interaction between biological, ecological and neuropsychological causal factors. Research has attempted to determine what neurological deficits or anomalies underpin sexual violence. Unfortunately, as with many past lines of research, sexual interest/preference has been conflated with criminal offending. In their review, Tenbergen et al. (2015) attempted to address this by considering pedophilia and evidence for the underlying alterations of structure and function in frontal, temporal, and limbic brain areas. They identify elevated rates of head injury and other previously identified markers of neurological perturbation that suggest brain injury may have contributed to the development of atypical sexual preference. Looking at research that incorporates the offending confound, research has found that compared to non-offending pedophiles, offending pedophiles exhibited inferior inhibitory control and moral decision making, characterized by prefrontal disturbances (Kärgel et al., 2017).

As seen in the ITSO, various factors interact to impact the neuropsychological functions that produce human behavior. Cantor et al. (2008) and Cantor (2013) demonstrated that there may be neurological vulnerabilities that exist within individuals that engage in sexual offending. Specifically, negative correlations were found between white matter volumes of the temporal and parietal lobes bilaterally in men diagnosed with pedophilia. As these regions of the brain connect the cortical regions that respond to sexual cues, the research suggests that pedophilia may be a result of partial disconnection within the network that recognizes sexually relevant stimuli (Cantor et al., 2008).

While evidence exists for brain injury and problematic sexual interest and behavior, it can also result in serious and disabling neuropsychiatric disorders such as cognitive deficits, personality change, and severe and chronic psychosis (Zhang and Sachdev, 2003). Specifically, research has suggested a shared vulnerability with psychosis/schizophrenia in the pedophilia population (Schiltz et al., 2007). Other research points to an acceleration effect such that men with a predisposition to schizophrenia engaged in sexual offending post-brain injury (Sachdev, 2001). Relatedly, individuals diagnosed with bipolar disorder, and who had sexually offended, were found to have higher rates of past brain injury (DelBello et al., 1999).

This paper summarizes two small studies that attempted to characterize the relationship between psychosis and problematic sexual interests and behavior. The first study sampled from a group of individuals diagnosed with a psychotic illness to describe their sexually-themed symptoms (i.e., sexual psychosis), related behaviors, and comorbidities. It was hypothesized that those who engaged in PSB would be more likely to exhibit sexual psychosis. The second study arose from preliminary analyses in study 1 which revealed higher than expected rates of brain injury within this group. Based on this observation, both studies included exploratory analyses regarding the relationship between brain injury and PSB among individuals with MMI.

## Study 1

### Methods

#### Participants

A retrospective file review was completed for 109 medical and medico-legal files. Information was gathered from both inpatient (*n* = 35) and outpatient (*n* = 74) civil and forensic psychiatric clinics at a large Canadian hospital.

#### Materials

Medical records included documentation such as psychiatric notes, psychological assessments, social work assessments, medico-legal assessments, medical consultation notes, and nursing notes. From these sources a number of variables were coded across the following broad domains: psychiatric and medical history, substance abuse history, criminal history, psychosocial history, prenatal/birthing history.

#### Procedures

Data abstraction was guided by a coding form prepared by the first author. Complete medical records were reviewed and data were coded by three trained clinical and research staff under the supervision of the first author. Training cases were completed together, and then two additional cases were parallel coded alongside the first author. Questions were addressed in regular meetings, and discrepancies were resolved by consensus and guided by the first author. Select cases were randomly reviewed to correct drift and ensure reliability. Interrater reliability, based on 12 cases, was very good, such that the average kappa coefficient for categorical variables was 0.86. The average intraclass correlation coefficient for continuous variables was 0.94. Data were analyzed using the Statistical Package for the Social Sciences version 25. This study was approved by the institutional research ethics board (#1503).

### Results

#### Sample Description

Participants were mostly single (64%) males (80%), with an average age of 44 years (range = 22–82 years). Most of the sample were unemployed at the time of coding (89%), and only 29% had completed high school. Clinically, most individuals had a primary diagnosis of schizophrenia or other psychotic disorder (88%), but this was to be expected based on the sampling criteria. Comorbidity was the rule, with 72% of the sample diagnosed with a substance use disorder, and 46% of the sample diagnosed with additional psychiatric conditions, such as mood disorders, anxiety disorders, and personality disorders. Rates of brain injury were high (28%), whereas only 3% of the sample had a formal paraphilia diagnosis. See Table 1 for a summary of both study results.

**TABLE 1:** Summary of participant information by study.

		Study 1	Study 2
Sex (male)		80%	86%
**Diagnoses**			
	Psychotic disorder	88%	83%
	Substance use disorder	72%	57%
	Brain injury (unspecified)	28%	2.5%
	Paraphilic disorder	3%	5%
**Family life**			
	Family history of mental illness	45%	40%
	Family history of substance abuse	36%	27%
	Family history of criminal activity	12%	7%
	Any history of trauma	34%	55%
**Criminal history**			
	History of general offending	73%	56%
	History of violent offending	64%	26%
	History of sexual offending	7%	18%

In terms of psychosocial or background information, most individuals were raised by both biological parents. A large proportion of participants’ files revealed a family history of mental illness (45%), substance abuse (36%), criminal activity (12%) and sexual behavior problems (7%). History of trauma was also reviewed, and 34% of the sample was found to have experienced at least one type of abuse, including physical (20%), verbal/emotional (20%), and sexual (16%) abuse.

Regardless of the clinic from which the sample was drawn, evidence of criminal activity was common (73%). More specifically, non-violent or general offending was noted in 54% of cases, whereas violent offending was documented in 64% of cases. Only 7% of cases were of a sexual nature, and there were no differences between the samples.

#### Sexual Psychosis: Nature and Prevalence

Across the entire sample 55% of individuals had documented sexualized thinking or behaviors (which need not be problematic or pathological). This was coded as a baseline for comparison to entries that were specifically identified as psychotic. By comparison, in 45% of cases the nature of psychotic symptoms were sexually themed to some extent, which we have defined as sexual psychosis (see Moulden and Marshall, 2017). Specifically, delusions of a sexual nature included themes of pedophilia, gender identity and sexual orientation, erotomania, jealousy/infidelity, delusions of reference (e.g., colors signify sexual arousal or interest), sexual abuse/exploitation, and genitalia. In terms of hallucinations, of the 60 individuals for which sexual psychosis was noted, auditory hallucinations were most common (80%) and included sexually arousing commentary, comments about sex, sexuality, or abuse, and endorsements of delusional beliefs. In 20% of these cases, visual hallucination were documented (e.g., naked shadows).

#### Problematic Sexual Behavior

From the documentation, 33 files included evidence of PSB, regardless of the setting from which the case was drawn (i.e., both forensic and non-forensic). Problematic sexual behavior is the term used to describe all types of inappropriate behavior, including boundary violations and illegal conduct, even if it was not pursued criminally. Inappropriate sexual comments (48%) were most common, followed by inappropriate sexual interactions (39%), and public nudity/masturbation (36%). Less frequent, but more concerning were incidents of child sexual abuse (15%), sexual assault (18%), and threats of sexual violence (12%). Sexual psychosis was reported in 70% of cases with PSB. In 15% of cases the PSB was known to be a direct result of sexual psychosis, including command hallucinations, or delusional. In addition to the disproportionate rates of sexual psychosis among those who had engaged in PSB, rates of brain injury in this group were remarkable (48%). In fact, individuals exhibiting PSB were 3.83 times more likely to have a history of brain injury, but not developmental or intellectual disorders.

## Study 2

### Methods

#### Participants

A retrospective file review was completed for 1,240 individuals in the forensic psychiatric system in Ontario, Canada, representing all active cases under the jurisdiction of the Ontario Review Board (ORB) between 2014 and 2015^1^. The Review Boards are independent tribunals mandated under the *Criminal Code of Canada*. While under federal legislation review boards fall under the jurisdiction of provincial/territorial healthcare ministries. Any individual found to be not criminally responsible or unfit to stand trial falls under the purview of these tribunals.

#### Materials

Data were retrieved from annual hospital reports prepared for the Ontario Review Board. These reports represent a detailed summary of the individual’s year based on input from members of the entire clinical team (e.g., nursing, psychiatry, psychology, social work, occupational therapy, recreation therapy, etc.). The report is cumulative, such that each year, the past review period summaries are retained in the document, and the most current clinical summary is added, resulting in a detailed, accurate, and complete compilation of the individual’s information and progress while under the ORB. Based on these reports data were coded for entry into a large database, including similar variables described in study 1: criminal history, psychiatric and medical history, substance abuse history, and psychosocial history.

#### Procedures

Study 2 was part of a larger project examining all forensic patients in Ontario, Canada. All research staff were trained by the senior investigators of that project, which included two of the authors (GC, HM). Coding materials and a coding manual were developed by the research team for the main project, who then trained alongside the research assistants before they were permitted to code independently. Approximately 10% of the files were coded by two raters to evaluate reliability. Excellent interrater reliability was achieved for the 10% of cases in the sample that were coded by two coders. The average kappa coefficient for categorical variables was 0.83. The average intraclass correlation coefficient for continuous variables was 0.92.

Coded variables included information about personal history, psychiatric history, criminal history, index offense characteristics, and risk assessment scores. Categories that were specifically coded using only information in the reporting year (2014–2015) included: forensic status, diagnoses, treatment, aggression, self-harm, and dispositions. When information on the aforementioned categories were discussed in the report (e.g., family history) but the presence of a specific variable was not mentioned (e.g., family history of mental illness) the item was coded as non-existent for the accused (e.g., ‘‘No’’ family history of mental illness). Categories that were completely missing from the report (e.g., no mention of family history in the report) were coded as ‘‘unknown’’ (e.g., family history of mental illness ‘‘unknown’’)^2^. All data were analyzed using the Statistical Package for the Social Sciences version 26. The current study obtained ethical approval from the institutional research ethics board (#8140).

## Results

The sample consisted of 1,063 males (86%), 173 females (14%), and 4 individuals who identified as transgender (0.3%). The median age at the time of the annual report was 41 years (range = 32–52 years). Not surprisingly, most individuals had a diagnosis of psychosis (83%), reflecting a common factor in the determination of criminal responsibility and fitness to stand trial. Similarly, schizophrenia was the most common primary diagnosis (57%). Psychiatric comorbidity was again the rule (75%), with 57% of individuals diagnosed with a substance abuse disorder, and 28% diagnosed with a personality disorder. The complex and enduring nature of forensic patients was exemplified by high rates of prior psychiatric admission (83%), and 81% had a history of psychiatric relapse following discharge from a mental health facility. Only 2.5% of this sample had a diagnosis of any brain injury, including acquired brain injury (0.3%), traumatic brain injury (0.4%) and other brain injury diagnoses (0.1%). A small proportion of the sample was diagnosed with a paraphilia (5%).

Personal history data revealed that most individuals were raised by both biological parents (77%). It was found that 40% of the sample had a family history of mental illness, 27% had a family history of substance abuse, and 7% had a family member with a criminal history. Over half of the cases included at least one adverse childhood experience (55%).

In terms of offense type, based on both criminal history and index offenses, 56% of participants were classified as general offenders (no formal history of any type of violence), 26% were classified as violent offenders (no formal history of any type of sexual violence), and 18% were classified as sexual offenders. Half (50%) of the sample had a history of inpatient physical aggression against others, followed by verbal aggression (34%), physical aggression against objects (30%), and sexual aggression (28%).

Of the individuals who had a history of a sexual offense, there was no significant difference in the odds of having a brain injury compared to non-sexual offenders (OR = 0.56). When the PSB definition was expanded to include both adjudicated and non-adjudicated sexual acts, such as inpatient sexual violence that was not processed criminally, there remained no effect for a brain injury (OR = 0.72).

## Discussion

The current findings describe emerging data in the relationship between brain injury, psychosis and problematic sexual behavior that may help explain the etiology and expression of PSB within this group. It is our aim to contribute to the much needed research on if and how mental health issues contribute to sexual offending, and most importantly the treatment and risk management for this select group.

Among individuals with psychotic illness, those exhibiting PSB appear to experience higher rates of sexually themed psychosis compared to individuals who do not engage in PSB. While this makes intuitive sense, given the small number of individuals with psychosis adjudicated for sexual offenses, even when the behaviors would likely be considered criminal in nature, this relationship has not previously been well established. Among individuals with MMI, such as schizophrenia, sexually themed psychotic symptoms are common, and appear to be related to problematic sexual behavior, and even sexual offending. Although these results are only preliminary, it seems that even without direct causal links as suggested by one typology (e.g., command hallucinations), psychotic symptoms are implicated still, perhaps because of the cognitive and behavioral disorganization and instability resulting in impulsive and disinhibited behavior. While there was evidence of personality disorders amongst the sample, this didn’t distinguish between groups. However, brain injury, and specifically, acquired injuries vs. congenital conditions, appeared to contribute a specific liability for PSB in the context of a psychotic illness.

This finding is consistent with two of the typologies initially described by Drake and Pathé (2004) and elaborated more recently in our work (Moulden and Marshall, 2017; Moulden et al., 2020), including the disinhibition and disorganization of psychosis, and neurological impairments. Of course, the mere presence of these symptoms does not mean that individuals with sexual psychosis are compelled to engage in PSB. However, it does support the notion that the acts may be congruent with, reflect a degree of preoccupation with, and may exacerbate features/negative symptoms of, psychotic illnesses (e.g., inhibition, theory of mind, and social competency) thereby indirectly contributing to the behavior.

Second, when examining the broader concept of PSB among both civil and forensic patients, as opposed to only formal criminal sanctions for sexual offending, a brain injury appeared to be a catalyst of such behavior among those with psychosis. The study 1 results support previous work that has hypothesized a shared vulnerability for psychosis and paraphilias or sexual offending for some individuals (Schiltz et al., 2007; Cantor, 2013). In both research areas brain injury, and particularly early head trauma, has correlated with the later onset of these illnesses. Although the mechanisms remain unclear, some potential candidates include neurochemical perturbance and anatomical and functional disruption (e.g., white matter connectivity; Tenbergen et al., 2015). However, this effect was lost when forensic only data were examined.

### Strengths, Limitations and Future Directions

This paper represents an attempt to (a) test hypotheses about the relationship between MMI and PSB and (b) contribute to the call for research by Mann and colleagues. Strengths of this research include the selected samples for MMI and the inclusion of both adjudicated and non-adjudicated PSB. Most research has understandably focused on justice-involved participants. However, often times socially deviant behavior committed in the context of a civil psychiatric admission or by individuals in residential care goes undetected/unreported. This is also the case for forensic patients, who despite reoffending are not formally charged, because “they are already in the system.” The different samples and associated data also allowed us to examine questions regarding the content of symptoms, and comorbidities which have not been readily considered, despite anecdotal clinical evidence, such as brain injury.

On the other hand, the nature of the design precluded claims of causation and cannot help explain reoffence and risk as a fundamental part in the determination of psychologically meaningful risk factors. However, this would be somewhat premature given the methodological issues that remain unsettled, such as how reoffence would be defined. Furthermore, the samples were of convenience, and based on retrospective data which limits variables of interest, comparison between samples, and ultimately the types of questions to be asked. Use of these retrospective sources means our study was hampered by different variables, missing or unknown data, such that only data recorded as a matter of course or deemed relevant made its way into the records, which were themselves different in nature (e.g., educational achievement). For example, given the drastically different rates of brain injury between the two samples one wonders if it was underreported among cases in Study 2, particularly as secondary or tertiary diagnoses. Whereas in the first sample, the entire medical record was reviewed instead of a summary report. An additional limitation with respect to the brain injury data was the missing date of injury. Although all diagnoses were before the current hospitalization, the precise relationship to the expression of PSB could not be determined. To address this issue, it would be worthwhile to examine the role of brain injury in PSB with an independent sample in which these specific questions could be incorporated into the study design prospectively.

An additional consideration is the role of confounding factors, which could account for the observed effects. For example, over half of the second sample had experienced childhood psychological adversity, such as abuse, parental separation, or other family hardship such as substance abuse or incarceration. Disproportionately high rates of adversity have been found among Individuals who engage in sexual offending (Levenson et al., 2014) and this has been replicated within forensic groups as well, who are defined by the presence of MMI (Stinson et al., 2021). Although these findings are non-specific regarding the types of abuse and the mechanisms by which they affect subsequent mental health and behavior, one pathway may be that early neural alterations result in some of the issues with connectivity, neurochemistry, or functionality in later life. More research examining the underlying mechanisms between early childhood adversity, psychiatric illness, such as psychosis and paraphilic disorders, and offending behavior may help differentiate and explain the route.

### Research and Clinical Implications

There is much work to be done in the area of MMI and PSB. Prospective research that follows individuals with vulnerabilities for MMI can help address questions of causation with respect to onset of illness and PSB, and the degree to which brain injury potentiates the effect. Imaging research is important to help clarify the area(s) of concern, be it anatomical, connective, functional or chemical. All are currently contenders, and as suggested by researchers in the area, it is likely interactions that produce the behavior of concern.

At the outset, and in our other work, we have maintained that more important than the role of mental illness as a risk predictor, is its role as a non-criminogenic need or responsivity factor, and the implications of MMI for treatment and management. MMI may not be alleviated, but can be managed. It fits the description of propensity as defined by Mann et al. (2010), which untreated, can increase risk and undermine treatment. The current research suggests that offense specific treatment for individuals with psychosis needs to attend to symptoms of MMI, including the sexually themed psychosis, negative symptoms, and associated features, in addition to the risk factors known to predict sexual offending among those without MMI. Furthermore, if brain injury is disproportionately found among this group, there may be other factors to consider related to learning, memory, processing, and executive functioning that can impact both the offending itself and the way treatment is delivered (see Moulden and Marshall, 2017; Moulden et al., 2020).

With respect to risk assessment, MMI can influence the expression of risk factors and potentially elevate risk scores. For example, committed relationships or living with a lover, issues such as problem solving and coping, can be impacted by social and cognitive features of psychosis. While the presence or absence of a factor may not be dictated by MMI, keeping these issues in mind, can help assessors with risk formulation and risk scenario planning, so as to interpret and address risk in a more individualized way (Kelley and Thornton, 2015).

## Conclusion

Major mental illness represents a clear example of propensity as described by Mann and her colleagues in that it predicts observations of thoughts, emotion and behavior, but also the interaction between an individual and others around them. While clinically evident among those who work with this unique group, relatively little work has been done to develop and test the theoretical foundation for MMI as etiologically related to PSB in particular. This is despite neurological bases in broad integrated theories of sexual offending, as well as specific theories and typologies proposed for sexual offending in the context of MMI. Although much work remains to be done in order to robustly determine the true contribution of MMI to PSB, the current research provides some support for a potential underlying neurological vulnerability and greater understanding about the relationship between sexual psychosis and offending behavior.

## Data Availability Statement

The data analyzed in this study is subject to the following licenses/restrictions: Study 1 data were extracted from personal health information in medical records. Study 2 data were extracted from publicly available data from the Ontario Review Board. Requests to access these datasets should be directed to HM, hmoulden@stjoes.ca.

## Ethics Statement

The studies involving human participants were reviewed and approved by Hamilton Integrated Research Ethics Board. Written informed consent for participation was not required for this study in accordance with the national legislation and the institutional requirements.

## Author Contributions

HM: research question, design, data collection, analysis, and manuscript preparation. CM: data collection and analysis. AL: data analysis and manuscript preparation. GC: research question and manuscript preparation. All authors contributed to the article and approved the submitted version.

## Conflict of Interest

The authors declare that the research was conducted in the absence of any commercial or financial relationships that could be construed as a potential conflict of interest.

## Publisher’s Note

All claims expressed in this article are solely those of the authors and do not necessarily represent those of their affiliated organizations, or those of the publisher, the editors and the reviewers. Any product that may be evaluated in this article, or claim that may be made by its manufacturer, is not guaranteed or endorsed by the publisher.

## Footnotes

1 Hospital reports for accused awaiting their initial review board hearing, waiting to return to court, had been recently discharged from the ORB, or reports not entered into the electronic system were not received.

2 Reported results are corrected for missing or unknown data.
